# The impact of older adult care policy mixes on the construction of senior centers

**DOI:** 10.3389/fpubh.2023.1193070

**Published:** 2023-06-15

**Authors:** Xin Chen

**Affiliations:** School of Statistics and Applied Mathematics, Anhui University of Finance and Economics, Bengbu, China

**Keywords:** older adult care policy mix, comprehensiveness, consistency, balance, senior center

## Abstract

The problem of an aging population is becoming increasingly serious, and the establishment of senior centers helps to promote the physical health and mental health of the older adult, a key factor in achieving the high-quality development of the old-age security industry. The government has issued a number of policies to promote the establishment and development of senior centers. However, more and more older adult care policy mixes have gradually shown the phenomenon of poor policy connection, confusing standards, and even mutually exclusive content, resulting in many problems in the construction of policy-driven senior centers. Therefore, based on the overall perspective of the older adult care policy mix, this paper uses the GMM method to explore the impact of the comprehensiveness, balance, and consistency of older adult care policy tool portfolios issued by Chinese government agencies on the construction of senior centers in China. The empirical analysis results show that a comprehensive and consistent policy mix can promote the establishment of senior centers, while the balance of the policy mix will inhibit the establishment of senior centers. This paper analyzes the impact of older adult care policy on the construction of senior centers from the perspective of the policy mix, which helps to clarify the different policy effects produced by different policy mix characteristics and provides feasible policy suggestions for the government to formulate more reasonable and effective policies.

## 1. Introduction

As of the end of 2021, China’s older adult population aged 60 and above totaled 267.36 million, accounting for 18.9% of the total population. The national older adult population aged 65 and above is 200.56 million, accounting for 14.2% of the total population, and the older adult population is about to enter deep aging. In recent years, China’s aging rate has continued to rise. Compared with 2010, the aging rate has increased by 5.44 percentage points, and the phenomenon of social aging has become increasingly serious (1, 2). How to promote the physical and mental health development of the older adult has become an extremely important research question, and received widespread attention (3, 4). As an important aspect of the construction of older adult service facilities (2), the construction of senior centers can not only effectively meet the needs of the older adult for basic life, basic medical care, and basic security, but also provide cultural, friendship, entertainment, and leisure services. It is an important means of promoting the physical and mental health development of the older adult and ultimately the high-quality development of China’s older adult care service system (5, 6). An older adult care activity center refers to a special institution and place with a certain scale that provides comprehensive cultural and recreational activities for the older adult, and its functional core includes the following two parts: (1) entertainment and rest for the older adult and (2) auxiliary functions such as catering, medical care, management, and other aspects (7). Building more senior centers can help promote the physical health and mental health of the older adult, help to cope with the challenges of aging, and achieve healthy aging (8).

The construction of senior centers requires much resources, and there are problems such as high upfront investment costs and the time it takes to see a return on funding (4, 9). In addition, the public has insufficient confidence in the social pension industry. This has led to an over-reliance on market investment resources to build senior centers. These resources are not sufficient to meet China’s growing demand for older adult care services (10, 11). Therefore, to accelerate the development of the physical and mental health of the older adult and the high-quality development of the older adult service system, the Chinese government has successively promulgated a series of relevant policies for the older adult, such as financial subsidies, tax exemptions, and standardized construction standards (2). These policies promote the construction of senior centers. However, the result of the promulgation of a large number of policies related to the older adult is that policies issued by governments at all levels have formed a combination of older adult care policies. The problems of large policy range, inconsistent goals, and ambiguous planning in the combination of older adult care policies are not conducive to promoting the flow of social resources to the construction of senior centers. In summary, in order to accelerate the construction of senior centers and promote the construction of an age-friendly society, it is necessary to explore the influence mechanism of the characteristics of the existing older adult care policy mix on the construction of senior centers, to help government departments formulate more accurate and effective policies, and ultimately achieve the high-quality development of China’s older adult care service system.

Thus, in order to better promote high-quality living for the older adult, this paper aims to give useful policy suggestions to policy makers by analyzing the policies related to older adult care services promulgated by the State Council and various ministries and commissions of the State Council from 2011 to 2019. This paper explores the specific impact of the comprehensiveness, balance, and consistency of the older adult care policy mix on the construction of senior centers in various regions of China. Following prior research, we divided the older adult care policy mix instrument into the following three categories: supply-based, demand-based, and environmental-based older adult care service policy instruments (2, 12). Then, we built statistical models to investigate the influence of policy mix characteristics on the construction of senior centers. The empirical analysis results show that the comprehensiveness and consistency of the older adult care policy mix can promote the construction of the senior centers, while the balance of the older adult care policy mix may inhibit the construction of the senior centers.

Our paper makes several contributions. The research conclusions of this paper are helpful in clarifying the effectiveness of China’s existing older adult care policies, identifying the aspects to be improved, and providing a theoretical basis for government departments to formulate more accurate and effective policies in the future, and then give full play to the combination of policy tools. However, in the existing policy mix, there is an interaction between a single policy, and there are differences in the impact of the comprehensiveness, consistency, and balance of the older adult care policy mix on the construction of senior centers. The characteristics of the policies play significant roles in promoting the high-quality development of China’s old-age service system.

## 2. Literature review and hypothesis

### 2.1. Older adult care policy mix literature

For China, in the context of the transition economy, the role of the government in promoting old-age security is extremely critical, and the old-age-related policies formulated by government agencies will affect the resource investment of social organizations in the old-age security industry, such as the establishment of more senior centers, the provision of more beds, and the establishment of more business organizations aimed at improving the quality of life of the older adult (2, 13). Studies have pointed out that old-age policies will have an impact on the resource investment of the old-age security industry. However, most of the existing studies regard older adult care policies as homogeneous policies, ignoring the great differences between older adult care policies. Multiple older adult care policies together form an older adult care policy mix, and there are often interactions between multiple policies in the policy mix, and there are differences or even contradictions between policies, factors that have a differential impact on policy outcomes (14–16).

Scholars have proposed that the overall effect of different policy mixes is different, there are differences in policy mixes in pension resource investment in different regions, and the impact of the same policy mix in different regions is also different (2, 17). In the analysis of the effective impact of policy mix, scholars have pointed out that clarifying the characteristics of policy mix is a prerequisite for better understanding and evaluating the effect of a policy mix (18). The government’s older adult care policy mix is characterized by investment in older adult care resources and the construction of senior centers.

In order to deeply understand the characteristics of the older adult policy mix, it is important to describe the differences and nature of different policies. Existing literature has pointed out that comprehensiveness, balance, and consistency are the most important characteristics of the policy mix (19–21). These characteristics may play different roles in affecting the efficiency and effectiveness of the policy mix.

However, there is little research on the impact of older adult care policy on old-age security in the existing policy mix literature, and there is a lack of analysis of the characteristics of the policy mix, such as the impact of the most important policy mix on the investment of pension resources. Clarifying the effects of different characteristics of the older adult policy mix on older adult resource input can help make related policies better, thereby improving the quality of old-age security.

### 2.2. Hypothesis

Policy tools are the practical means and methods adopted by policymakers to achieve set policy goals. Some scholars have pointed out that policy mix refers to the combination of different policy instruments, and the interaction between policy instruments is the basis of policy mix. Some scholars have classified policy tools into the following four categories: regulatory instruments, economic instruments, financial instruments, and soft instruments (22). Other scholars divide the policy mix instruments into categories such as mandatory, market, information transmission, and voluntary (23). In our study, when dividing the older adult policy mix, we follow the framework proposed by Rothwell and Zegveld (12). We divide the policy mix instruments into environmental, supply-based, and demand-oriented policy instruments (2, 24). Supply-based policy instruments include effective support in terms of funds, facilities, technology and information. Demand-oriented policy instruments include service outsourcing, direct procurement, international exchanges, and trade regulation. Environmental policy instruments can provide a favorable policy environment through using tax incentives, regulatory controls and strategic measures.

Based on the older adult care policies collected in this paper, and on the results of the text-mining method, we further analyzed the diversified older adult care policy tools involved in multiple older adult care policies. The specific content involved in environmental policy tools, supply-oriented policy tools, and demand-oriented policy tools were further analyzed, and the keywords were analyzed and identified separately. Based on Zhang’s (2) research, Table 1 shows the specific classification of older adult care policy tools and the description of content keywords.

**Table 1:** Classification of policy instruments and description of their contents.

Type of policy instrument	Content	Keyword
Demand Policy	Government procurement	The government buys equipment, services
	Service outsourcing	Social capital
	Nurturing the market	Market development, growth
	International demand	Enhance international cooperation and exchanges
Supply policy	Talent development	Education and training, project investment
	Capital investment	Funding, resources, and capital investment
	Technology investment	Technology research and development, innovation
	Facility input	Supporting facilities, aging transformation
	Information Services	Digital platform, information exchange
Environmental Policy	Tax benefits	Tax deductions
	Technical support	Industry–university research cooperation, alliance cooperation
	Land policy	Venue rent reduction
	Administrative measures	Streamline the approval process
	Other economic policies	Utility fee waiver

#### 2.2.1. The influence of comprehensive older adult care policy mixes on the number of senior centers

The comprehensive combination of older adult care policies measures the breadth of policy application. Therefore, to promote the effectiveness of pension security, a variety of policy instruments will be used comprehensively. The comprehensive measurement of the comprehensiveness and diversity of older adult care policy tools, and the combination of policies have a degree of complexity. The use of a wide range of policies can more effectively activate policy departments at all levels and can incentivize other organizations to contribute to the security of the older adult, helping to provide a good environment for promoting the physical and mental health of the older adult. The stronger the comprehensiveness, the more types of policy tools involved in the older adult care policy mix, and the more detailed the policy objectives formulated.

For example, in terms of the comprehensive use of older adult care policy tools, the government has issued a series of policies to promote the development of the pension security industry, such as promoting the introduction of pension equipment, platforms, system construction, pension institution construction incentives, tax exemptions, and other policy mixes. The improvement in the breadth of the older adult care policy mix can promote the development of the pension security industry and the promotion of the high-quality development of the care of the older adult to provide more favorable conditions.

In short, adopting a variety of policy tools can improve policy effectiveness. The government can accelerate the investment of more resources in the construction of older adult care centers by combining multiple tools in society (25), thereby improving the promotion of the healthy development of the older adult care industry. Zhang’s (2) research also points out that the comprehensive combination of older adult care policies can promote the growth of the number of older adult care resource beds. Therefore, we propose the following hypothesis:

*Hypothesis 1*: The comprehensiveness of policy tools (demand, supply, environment) of older adult care policy can positively promote the number of senior centers.

The balance of the older adult care policy mix measures the difference between the intensity and development level of different policies in the older adult care policy mix (26). The balance of intensity and development level between policies is conducive to promoting the healthy development of old-age security. The implementation of a balanced policy mix helps to form a more reliable and stable policy framework, which helps to enhance the confidence of social organizations in the investment of the older adult care industry, thereby promoting a willingness to build more activity centers for the older adult. Conversely, the use of only one policy tool often cannot meet the complex social environment needed to promote the investment of pension resources into the construction of senior centers. If it were possible to combine the advantages of a variety of different types of policy tools, while avoiding their disadvantages, it would greatly enhance the policy effect.

However, unlike the demands of eating, drinking, and medical treatment to meet the basic living security of the older adult, the purpose of the construction of a senior center is to promote the physical and mental health of the older adult and to simultaneously promote the healthy development of the older adult (27). If the older adult care policy is too balanced and there is no special attention paid to the construction of the senior center, then under normal circumstances, the pension resources are more likely to be allocated to meet the basic needs of the older adult in terms of eating, drinking, and medical treatment. Therefore, the balance of policy mix is not conducive to the construction of senior centers. Therefore, we propose the following hypothesis:

*Hypothesis 2*: The balance of older adult care policy mix policy tools (demand, supply, environment) can positively promote the number of senior centers.

The consistency of the older adult care policy mix measures the synergy between policies within the policy mix and reflects the differences between policies. There are fewer conflicts between policies within a highly consistent policy mix; there is even some synergy. The consistency and coordination between policies can provide a good institutional environment for the investment of social pension resources and the construction of senior centers and can more effectively promote the high-quality development of the older adult care industry (28). The impact of a single science and technology policy on promoting old-age security is limited, so the government often promulgates a variety of policy tools or policy objectives to achieve a combination of different policies through coordinated allocation to give full play to the advantages of the policy mix more effectively. Coherence between policies in the policy mix can amplify policy effectiveness to a greater extent and promote the effectiveness and accessibility of care policies (29). For example, some policies emphasize that a variety of tax relief policies for the construction of senior centers will help promote their construction. Based on different demands, there are sometimes conflicts between policies formulated by different government agencies, thereby reducing the consistency of the older adult care policy mix and not being conducive to the construction of senior centers. Based on the above, we propose the following hypothesis:

*Hypothesis 3*: The consistency of older adult care policy mix policy tools (demand, supply, environment) can positively promote the number of senior centers.

## 3. Sample and methods

### 3.1. Data collection

This paper selects the relevant policy documents of the State Council and various ministries and commissions from 2011 to 2019 for quantitative analysis of their text. Following prior literature (2), the relevent policies are derived from the following sections, respectively as: official websites of the State Council, various ministries and commissions of the State Council, and the legal database of Peking University, the most recognized database in China. In order to screen out policies closely related to the research topic of this study, we only keep the policies that contain the following terms: “old-age security,” “home-based care,” “community care,” “medical and older adult care integration,” “pension system,” “smart old-age care,” “old-age service,” “Internet old-age care,” “aging,” and other related terms (2). To ensure the accuracy of text analysis, the process of text analysis was compared by members of the author’s team, and a unified conclusion was reached after a group discussion on any divergent parts. To ensure the accuracy of the selected older adult care policy samples, the members of the research group screened the pension-related policies separately. After the preliminary screening, they sorted out and confirmed the 2011–2019 policies through group discussion. The policies promulgated in the past few years to promote the development of China’s old-age security and its strong correlation.

### 3.2. Variable measures and model selection

#### 3.2.1. Variable measures

##### 3.2.1.1. Dependent variable

Number of senior centers (*SC*): a senior center is a public place dedicated to providing leisure, entertainment, and communication, and to cultivating the mood of the older adult. It is a special type of public building. Based on the pension model of home care and community action, it aims to meet the leisure and older adult care needs of older people. The data on the number of older adult activity stations/centers/rooms in this article are derived from the China Stock Market & Accounting Research Database (CSMAR), the most recognized database in China.

##### 3.2.1.2. Argument

Based on the research of Rothwell and Zegveld (30), this paper focuses on the policy goal dimension of older adult care policy and subdivides policy tools into three categories based on the classification standards of the existing literature, namely demand, supply, and environment. Among them, the policy strength is divided into the following five categories: (1) Notices, announcements; (2) Provisional regulations, measures, opinions, and plans of various ministries and commissions; (3) Interim regulations and plans issued by the State Council and regulations of various ministries and commissions; (4) Regulations promulgated by the State Council and decrees issued by various ministries and commissions; (5) Laws promulgated by the National People’s Congress and its Standing Committee. Based on the quantitative analysis of older adult care policies, this paper measures the comprehensiveness, consistency, and balance of different policy types in the same dimension and different policy indicator combinations in the same policy type of the government’s old-age security policies promulgated from 2011 to 2019. When calculating the eigenvalues of the combination of science and technology policy objectives and policy tools, the average value of the sum of the indicators under the policy objective or tool dimension was used to represent its eigenvalue score.

The formula for calculating the policy mix is as follows:


(1) $$TS_{t}=\overset{N}{\underset{j=1}{\sum }}{\mathrm{Score}}_{tj}\times P_{tj}\;t\;\;\;\;\in \left[2017,2019\right]$$

(2) $$POL=\overset{l}{\underset{r=1}{\sum }}TS_{t}^{r}$$

In the above formula, t refers to the year, j represents any science and technology policy promulgated in year t, N is the sum of science and technology policies promulgated in year t, and Score measures the index score of the policy. P reflects the effectiveness of the policy, and TST represents the indicator characteristics of the science and technology policy promulgated by the government in year t. r represents policy indicators, and l is the total number of policy indicators within the same policy type.

When measuring the consistency of the science and technology policy mix, the policy indicator vector is constructed based on the policy indicators. The cosine value of the average vector angle is measured by analyzing the policy text. The consistency of the policy mix is measured, and the calculation $X_{t}^{i}=\left(x_{t}^{i1},x_{t}^{i2},\ldots ,x_{t}^{il}\right)$ formula is as follows:


(3) $$POL=\left(\overset{l}{\underset{r=1}{\sum }}TS\right)\times \frac{\overset{k}{\underset{i=1}{\sum }}\overset{k}{\underset{j=1}{\sum }}\cos\left(X_{t}^{i},X_{t}^{j}\right)}{N\times \left(N-1\right)/2}$$

(4) $$\cos\left(X_{t}^{i},X_{t}^{j}\right)=\frac{\overset{l}{\underset{r=1}{\sum }}\left(x_{t}^{ir}\times x_{t}^{jr}\right)}{{\sqrt{\overset{l}{\underset{r=1}{\sum }}{\left(x_{t}^{ir}\right)}^{2}}}^{*}\sqrt{\overset{l}{\underset{r=1}{\sum }}{\left(x_{t}^{jr}\right)}^{2}}},\forall i\neq j$$

In the above formula, i, j refers to any two science and technology policies issued by the government in year t, which represents the vector angle cosine value between policy i and $\cos\left(x_{t}^{i},x_{t}^{j}\right)$ j. The value reflects the consistency between policies.

When measuring the balance of the science and technology policy mix, the correlation index between the policy indicators is first measured, and then the balance of the policy mix is measured by calculating the standard deviation of the correlation index. The calculation formula is as follows:


(5) $$Pol_{t}^{d}={\left[\frac{\left|TS_{mt}-TS_{nt}\right|}{\sqrt{TS_{mt}+TS_{nt}}}\right]}^{-1},\forall m\neq n$$

(6) $$TolPol_{t}={\sqrt{\frac{\overset{n^{*}}{\underset{d=1}{\sum }}\left(Pol_{t}^{d}-\frac{\overset{n^{*}}{\underset{d=1}{\sum }}Pol_{t}^{d}}{n^{*}}\right)}{n^{*}}}}^{-1}$$

In the above formula, m and n represent different policy indicators, and d refers to the combination of policy indicators. $n^{*}$ indicates the sum of two combinations of science and technology policy indicators.

##### 3.2.1.3. Control variables

This paper controls for factors that may affect the number of senior centers and introduces the following control variables: (1) Pension income (abbreviated as *PI*). This reflects the pension level of each region. The higher the pension income, the stronger the ability to build senior centers, (2) Pension outlays *(PO)*, reflecting the level of fund expenditure in each region in terms of pension security. The higher the value, the more attention the region attaches to the guarantee of pension services, (3) GDP *per capita* (*PG*) GDP *per capita* reflects socioeconomic development. The higher its value, the more resources will be available to invest in the number of centers for the older adult, (4) Fixed assets investment (*FI*), reflecting the strength of resources invested in social construction in various regions of China. The more fixed asset investment is made, the more resources can be used to build senior centers, (5) Old-age dependency ratio (OR). The higher the proportion of older adult support, the higher the demand for senior centers, and the greater the possibility of society investing resources in senior centers, thia variable is derived from CSMAR, and (6) Education level (EL). Measured by the proportion of general college graduates to the total population, the higher the level of social education, the more requirements for the physical and mental health of the older adult, and the higher the possibility of investing in the construction of senior centers.

### 3.3. Model selection

In order to solve the potential endogeneity, we employed a two-step system GMM regression method to investigate the effect of the older adult care policy mix on the construction of senior centers (31, 32). This method can handle endogenous regressors and is specifically suitable for the use of predetermined but not strictly exogenous regressors (33). The two-step system GMM can better solve weak instrument problems (32) and handle endogenous regressors. It is beneficial to the use of predetermined but not strictly exogenous regressors (33). The full model was established using the following equation:


$$\begin{array}{c} \mathrm{SCit}=\beta \mathrm{1 EPMit}+\beta \mathrm{2 EPMit}-1+\beta 3\;\mathrm{PI}\;\mathrm{it}+\beta \mathrm{4 POit}\\ +\beta \mathrm{3 PGit}+\beta 3\;\mathrm{FI}\;\mathrm{it}+\beta \mathrm{3 ORit}+\beta \mathrm{3 ELit}+\varepsilon it \end{array}$$

### 3.4. Results

Table 2 shows the means, standard deviations, and bivariate correlations of the variables. The correlation coefficient is a statistical indicator originally designed by Karl Pearson, which measures the degree of linear correlation between variables (34). To determine whether there were potential multicollinearity issues, variance inflation factors (VIF) of variables in the model were tested. All the VIFs were far lower than 10, which suggest that multicollinearity was not a significant issue (35).

**Table 2:** Means, standard deviations, and bivariate correlations of variables.

	Variables	Mean	Std. Dev.	1	2	3	4	5	6	7	8	9	
1	SC	10884.53	11728.71	1.000									
2	Comprehensiveness	114.6667	73.95074	−0.084	1.000								
3	Balance	1.218002	0.3333793	−0.044	−0.421***	1.000							
4	Consistency	41.864	27.70725	−0.087	0.992***	−0.377***	1.000						
5	PI	1060.934	876.1212	0.357***	0.301***	−0.069	0.299***	1.000					
6	PO	923.1701	750.1287	0.334***	0.368***	−0.087	0.366***	0.953***	1.000				
7	FI	17056.03	12625.88	0.612***	0.234***	−0.128**	0.225***	0.671***	0.671***	1.000			
8	PG	54021.61	26225.68	0.020	0.263***	−0.078	0.261***	0.603***	0.557***	0.255***	1.000		
9	EL	0.2641542	0.1016133	−0.172***	0.180***	−0.107*	0.172***	0.286***	0.307***	0.034	0.649***	1.000	
10	OR	13.8324	3.408956	0.218***	0.367***	−0.091	0.366***	0.564***	0.681***	0.544***	0.305***	0.319***	1.000

*n* = 2,884 for mean and standard deviation. Correlations ≥ 0.06 are significant at the 0.001 level. *t* statistics in parentheses, *p<0.05, **p<0.01, ***p<0.001.

#### 3.4.1. The impact of different policy tool combinations on the supply of older adult care service resources

This section analyzes the impact of the comprehensiveness, consistency, and balance of older adult care policy tools on the construction of senior centers. Table 2 presents the regression results predicting the effects of the older people policy mix on the establishments for senior centers. Model 1 suggests that the comprehensive older adult care policy mix tool will positively promote the construction of senior centers (*β* = 0 22, *p* < 0.01). This paper verifies the hypothesis 1 proposed in this paper, indicating that the increase in the breadth of the government’s pension-related policy mix and the strengthening of synergy between policies will promote the construction of older adult care centers.

Based on the results in Model 2, unlike our hypothesis 2, it appears that the balance of older adult care policy mix tools negatively inhibits the construction of senior centers (*β* = −3043.60, *p* < 0.01). This shows that the more balanced the development of the indicators of supply policy, demand policy, and environmental policy in the older adult care policy tools, the more detrimental it will be to the construction of senior centers.

Model 3 presents the effects of the older adult care policy mix tool consistency promoting the construction of senior centers (*β* = 20.44, *p* < 0.01). This verifies the hypothesis 3 put forward in this paper and proves that when the degree of similarity between the older adult care policies proposed by the government increases, it will help the resource input of the construction of the older adult care activity center.

Based on the results, the residuals of all models were significantly first-order autocorrelational at the 5% level. There was no second-order autocorrelation, and none of the Sargan statistics were significant. This shows that the instrumental variables used in each model are valid (Table 3).

**Table 3:** The effects of older adult policy mix on senior centers.

SC	Model 1	Model 2	Model 3
L.SC	0.22*** (37.58)	0.19*** (38.22)	0.22*** (35.98)
Comprehensiveness	7.51*** (16.40)		
Balance		−3043.60*** (−54.57)	
Consistency			20.44*** (16.61)
PI	0.21 (0.70)	0.16 (0.79)	0.55** (2.60)
PO	0.26 (0.65)	0.89** (3.29)	−0.25 (−1.00)
FI	0.13*** (13.59)	0.01 (0.87)	0.14*** (13.02)
PG	−0.10*** (−13.60)	−0.07*** (−12.62)	−0.11*** (−14.15)
EL	18045.05*** (26.77)	10876.84*** (10.79)	19617.04*** (18.47)
OR	−1352.81*** (−43.99)	−1178.39*** (−26.81)	−1355.95*** (−43.67)
Constant	22039.97*** (22.01)	26434.36*** (24.01)	22366.47*** (23.50)
Sargan	22.90	26.06	25.35
sarganp	(0.69)	(0.52)	(0.55)
arm1	−1.56	−1.87	−1.53
ar1p	(0.12)	(0.06)	(0.13)
arm2	1.56	1.54	1.59
ar2p	(1.88)	(1.88)	(1.89)
*N*	216	216	216

*t* statistics in parentheses, * *p* < 0.05, ** *p* < 0.01, *** *p* < 0.001.

#### 3.4.2. The impact of policy mix within the same policy instrument on the construction of senior centers

In this paper, the older adult care policy tools are divided into three types: demand policy, supply policy, and environmental policy. The impact of the comprehensiveness, consistency, and balance of these three policy tools on the construction of senior centers is analyzed, and Table 4 shows the results.

**Table 4:** The impact of the older adult care policy mixes on the construction of senior centers.

SC	Model 1	Model 2	Model 3	Model 4	Model 5	Model 6	Model 7	Model 8	Model 9
L. SC	0.22*** (37.58)	0.19*** (38.22)	0.22*** (35.98)	0.21*** (40.16)	0.18*** (48.20)	0.19*** (39.42)	0.22*** (38.32)	0.20*** (26.43)	0.21*** (42.62)
Comprehensiveness	7.51*** (16.40)			53.44*** (41.05)			23.69*** (25.62)		
Balance		−3043.60*** (−54.57)			−731.43*** (−59.73)			−498.53*** (−9.15)	
Consistency			20.44*** (16.61)			64.99*** (51.74)			48.61*** (20.03)
PI	0.21 (0.70)	0.16 (0.79)	0.55** (2.60)	0.98*** (3.77)	0.47** (3.20)	0.44 (1.48)	0.72* (2.27)	−0.03 (−0.11)	0.47 (1.42)
PO	0.26 (0.65)	0.89** (3.29)	−0.25 (−1.00)	−0.52 (−1.61)	1.11*** (3.56)	0.02 (0.04)	−0.25 (−0.58)	0.72* (2.13)	−0.18 (−0.38)
FI	0.13*** (13.59)	0.01 (0.87)	0.14*** (13.02)	0.12*** (11.15)	0.03*** (3.70)	0.10*** (13.61)	0.13*** (11.64)	0.10*** (5.40)	0.14*** (12.68)
PG	−0.10*** (−13.60)	−0.07*** (−12.62)	−0.11*** (−14.15)	−0.10*** (−23.97)	−0.04*** (−5.53)	−0.09*** (−18.90)	−0.11*** (−22.35)	−0.10*** (−21.94)	−0.11*** (−24.69)
EL	18045.05*** (26.77)	10876.84*** (10.79)	19617.04*** (18.47)	14956.65*** (26.77)	10660.80** (2.81)	13687.97*** (19.62)	17214.56*** (16.65)	17427.48*** (11.70)	18079.32*** (18.05)
OR	−1352.81*** (−43.99)	−1178.39*** (−26.81)	−1355.95*** (−43.67)	−1350.06*** (−47.27)	−1157.07*** (−25.28)	−1325.20*** (−68.47)	−1335.35*** (−53.17)	−1252.89*** (−36.47)	−1334.84*** (−60.08)
Constant	22039.97*** (22.01)	26434.36*** (24.01)	22366.47*** (23.50)	23415.25*** (23.73)	23389.32*** (15.55)	22474.26*** (21.11)	22329.24*** (23.19)	23661.03*** (24.21)	22335.15*** (23.23)
Sargan	22.90	26.06	25.35	25.33	27.23	21.52	24.36	26.09	23.84
sarganp	(0.69)	(0.52)	(0.55)	(0.56)	(0.45)	(0.76)	(0.61)	(0.51)	(0.64)
arm1	−1.56	−1.87	−1.53	−1.58	−1.83	−1.65	−1.56	−1.66	−1.56
ar1p	(0.12)	(0.06)	(0.13)	(0.11)	(0.07)	(0.10)	(0.12)	(0.10)	(0.12)
arm2	1.56	1.54	1.59	1.48	1.56	1.51	1.54	1.60	1.59
ar2p	(1.88)	(1.88)	(1.89)	(1.86)	(1.88)	(1.87)	(1.88)	(1.89)	(1.89)
N	216	216	216	216	216	216	216	216	216

*p* values in parentheses. Significant at 1%, ** at 5%, * at 10%. The level is significant. *t* statistics in parentheses, *p<0.05, **p<0.01, ***p<0.001.

The data analysis results in Table 4 show that from the perspective of the policy tools, as proposed above, the synthesis of demand policy, supply policy, and environmental policy can positively promote the construction of senior centers, indicating that the higher the level of policy promulgation departments in each sub-dimension of policy tools and the wider the scope of policy formulation, the more significant the promotion effect on the construction of senior centers. Second, the balance of demand policy, supply policy, and environmental policy has a negative impact on the construction of senior centers, indicating that the more balanced the setting of the internal indicators of each policy tool, the more even the development of the older adult service system. Finally, the consistency of demand policy, supply policy, and environmental policy will promote the construction of senior centers, because the consistency between policies will enhance the confidence of social resources in investing in older adult care centers. It will be more conducive to the construction and operation of older adult care institutions, thereby helping to promote the investment of pension resources in the building of senior centers.

## 4. Conclusions and countermeasures

### 4.1. Conclusion

This paper quantitatively analyzed the policy documents related to old-age security issued by the State Council and various ministries and commissions from 2011 to 2019, exploring the impact of the characteristics of the old-age policy mix on the senior centers in various regions of China by establishing an empirical analysis model. The main conclusion of this study is that in order to promote the development of the pension security industry, the comprehensiveness and consistency of the older adult care policy mix tools issued by the government will significantly promote the construction of senior centers, and the balance of older adult care policy mixes will inhibit the resource input of older adult care activity center construction. When exploring the impact of different policy indicator combinations on the construction of senior centers in policy tools, the three policy types under policy tools, demand policy, supply policy, and environmental policy, comprehensiveness, consistency, and balance, will have an impact on the construction of the senior center. Among them, the comprehensiveness and consistency of the combination of demand policy, supply policy, and environmental policy will have a positive impact on the construction of senior centers, and the balance will significantly inhibit the resource construction of senior centers. We believe that the balance of the older adult care policy mix will have a negative impact on the construction of senior centers because unlike the needs for eating, drinking, and medical treatment to meet the basic living security of the older adult, the purpose of the construction of senior centers is to promote the physical and mental health of the older adult at the same time, and promote the healthy development of the older adult (27). If the older adult care policy is too balanced and there is no special attention paid to the construction of senior centers, then under normal circumstances, the pension resources will be more inclined to meet the basic needs of the older adult in terms of eating, drinking, and medical treatment. Therefore, the balance of policy mix is not conducive to the construction of senior centers.

### 4.2. Policy implications and recommendations

The increasing proportion of the population aged 65 or over has raised growing challenges to long-sustainable development of public finances across countries (4, 36). In recent years, the role of senior centers in promoting the physical and mental health of the older adult has become increasingly important. The construction of senior centers is not only affected by the market environment but also plays a very important role in the national political system and macro situation (37). The relevant policies issued by the central government and governments at all levels are important promoters encouraging various bodies in society to actively build senior centers under the premise of limited economic benefits. As one of the important macro-level factors promoting the smooth development of old-age security, the influence of the characteristics of old-age policy mixes on the construction of old-age activity centers was explored and was found to be of great significance.

First, according to the above research conclusions, the formulation of older adult care policies needs to be considered as a whole, rather than just considering the formulation of a single policy, because there is an interaction between every single policy, and there are differences in the impact of the comprehensive, consistent, and balanced combination of older adult care policies on the construction of senior centers.

Second, strengthening the social security of the older adult group should not only involve the physical health of the older adult, but should also include the clothing, food, housing, and transportation needs of the older adult. It is also necessary to pay attention to the mental health and cultural needs of the older adult, to provide home care services for the older adult with chronic diseases under the guidance and support of the government, and to offer convenient services for the older adult who are physically weak. Service providers should have the ability to provide medical nursing, psychological counseling, and other professional services for the older adult to promote their physical and mental health and ensure the construction of high-quality older adult care services for the older adult.

Third, the government should play a leading role in promoting the supply of older adult care services. It should also be noted that in addition to the government, a variety of other bodies, such as families, communities, and enterprises, have their part to play. In order to ultimately achieve the physical and mental health of the older adult, a variety of subjects should be called upon to invest in the supply of older adult care resources, and as a community with unique natural advantages in older adult care services, the government should focus on building a unified older adult care service system (38), helping older people achieve physical and mental health by promoting the number of activity centers for the older adult.

Fourth, the government may implement older adult care service policies based on regional environment and preferences for older adult care needs, and try to avoid one-size fits all older adult care service policies. Our government can improve the allocation of older adult care service resources and increase the utilization rate of older adult care service resources by taking suitable policy tools. In order to promote the comprehensive development of older adult care services and broaden the older adult care service market, the government can take measures as expanding the promotion of older adult care service policies, clarifying older adult care service standards, improving older adult care service information platforms and innovating older adult care service products.

Finally, the key to improving older adult care quality is to innovate, the government should pay more attention on innovation. Innovation in older adult care area can help integrate resources, and provide integrated maintenance services. This not only meets the needs of healthy older adult care, but also is a common trend in the supply reform and policy innovation of older adult care services in countries. Policy innovation is needed to support, guide, and supervise the reform of medical and older adult care integration, including cross departmental collaboration strategies, exploratory long-term care insurance coverage policies, and human resource reserve policies.

### 4.3. Limitations and future research

Our study has several limitations, which offer possibilities for future studies. First, this paper only investigates the impact of old-age related policy mixes on the construction of senior centers. There are many aspects not covered in this article that are closely related to promoting the physical and mental health of the older adult and the high-quality development of older adult care services. These aspects include the construction of pension systems, the provision of basic security for the older adult, and the standardization of nursing homes. Future studies could investigate these topics.

Second, this paper only discusses emerging economies such as China. The universality of the research conclusion remains to be verified. Future studies could apply this study to developed countries like the United States, France, and Japan.

Moreover, this study does not explore the influence mechanism of the characteristics of provincial and ministerial governments or policy mixes at different regional levels on the construction of senior centers. Future research could further clarify the impact of provincial and ministerial policy mix characteristics on the construction of older adult activity stations.

Finally, there is no consistent conclusion on the impact of comprehensiveness, consistency, and balance of the policy mix related to older adult care services on innovation. Future research could expand and supplement the results of quantitative analysis by combining case studies, thereby enhancing the credibility of research conclusions through mixed research methods. Future research could use questionnaire research to explore the impact of old-age-related policies on the construction of senior centers.

## Data availability statement

The original contributions presented in the study are included in the article/supplementary material, further inquiries can be directed to the corresponding author.

## Author contributions

XC: conceptualization, methodology, and validation. The author have read and agreed to the published version of the manuscript.

## Conflict of interest

The author declares that the research was conducted in the absence of any commercial or financial relationships that could be construed as a potential conflict of interest.

## Publisher’s note

All claims expressed in this article are solely those of the authors and do not necessarily represent those of their affiliated organizations, or those of the publisher, the editors and the reviewers. Any product that may be evaluated in this article, or claim that may be made by its manufacturer, is not guaranteed or endorsed by the publisher.
